# *Cryptococcus neoformans* Infection in the Central Nervous System: The Battle between Host and Pathogen

**DOI:** 10.3390/jof8101069

**Published:** 2022-10-12

**Authors:** Yanli Chen, Zoe W. Shi, Ashley B. Strickland, Meiqing Shi

**Affiliations:** 1 Division of Immunology, Virginia-Maryland College of Veterinary Medicine and Maryland Pathogen Research Institute, University of Maryland, College Park, MD 20742, USA; 2 Department of Chemistry and Biochemistry, University of Maryland, College Park, MD 20742, USA

**Keywords:** fungus, *Cryptococcus neoformans*, cryptococcosis, fungal pathogenesis, meningoencephalitis, central nervous system, fungal dissemination, brain invasion, Trojan horse, transcytosis, paracellular crossing, microglia, monocytes, macrophages, T cells, NK cells, cytokines, virulence factors, polysaccharide capsule, laccase, melanin, phospholipase B1, urease, chitin

## Abstract

*Cryptococcus neoformans* (*C. neoformans*) is a pathogenic fungus with a global distribution. Humans become infected by inhaling the fungus from the environment, and the fungus initially colonizes the lungs. If the immune system fails to contain *C. neoformans* in the lungs, the fungus can disseminate to the blood and invade the central nervous system, resulting in fatal meningoencephalitis particularly in immunocompromised individuals including HIV/AIDS patients. Following brain invasion, *C. neoformans* will encounter host defenses involving resident as well as recruited immune cells in the brain. To overcome host defenses, *C. neoformans* possesses multiple virulence factors capable of modulating immune responses. The outcome of the interactions between the host and *C. neoformans* will determine the disease progression. In this review, we describe the current understanding of how *C. neoformans* migrates to the brain across the blood–brain barrier, and how the host immune system responds to the invading organism in the brain. We will also discuss the virulence factors that *C. neoformans* uses to modulate host immune responses.

## 1. Introduction

*Cryptococcus neoformans* (*C. neoformans*) is an encapsulated pathogenic fungus that infects humans and animals [1]. The fungus exists in environments such as soil, trees, and bird droppings. Humans become infected through the inhalation of *C. neoformans* [2,3]. The fungal cells interact initially with alveolar macrophages in the lungs, which may lead to the phagocytosis of the organism. Macrophages may kill ingested *C. neoformans*; alternatively, fungal cells can survive or grow within the phagocytes, depending on the host’s immune status [4]. If *C. neoformans* is not contained in the lungs, the fungus can migrate to the bloodstream and cross the blood–brain barrier (BBB), leading to the infection of the central nervous system (CNS) and meningoencephalitis [1,2,3].

Most cases of cryptococcal meningoencephalitis emerge in immunocompromised patients, including patients with an HIV-infection and organ transplants, although, rarely, immunocompetent patients can also develop the illness [2,3]. Cryptococcal meningoencephalitis is fatal without timely medical care, which involves long-term treatment with amphotericin B, flucytosine, and fluconazole [5]. Even with successful therapy, survivors of this illness often develop neurologic deficits and other adverse effects [6,7]. It was estimated that this fungal disease affects nearly 223,100 people each year and leads to 181,000 deaths annually [8].

Cellular immune responses play an essential role in the clearance of a cryptococcal infection [9]. Inflammatory monocytes/macrophages, microglial cells, and antigen-specific T cells including CD4^+^ and CD8^+^ T cells are critically involved in host’s immune responses to *C. neoformans* infections [10,11]. Th1 immune responses, characterized by IFN-γ, and Th17 immune responses, characterized by IL-17A, mediate the protection against *C. neoformans* [9,12]. IFN-γ activates macrophages and promotes the classical activation of macrophages, which is correlated with fungal clearance [9,13]. In contrast, Th2 cytokines such as IL-4, IL-5, and IL-13 drive the alternative activation of macrophages, which is associated with disease progression [9,14].

*C. neoformans* has developed sophisticated mechanisms to enable pathogenesis by escaping host defense mechanisms and modulating immune responses [4]. Its polysaccharide capsule and melanin production are the major virulence factors of *C. neoformans* [15,16,17]. In addition, *C. neoformans* secretes a number of virulence-associated enzymes [18]. Those virulence factors are critically involved in *C. neoformans’s* invasion into the CNS and its suppression of host immune responses. The outcome of the interaction between the host and *C. neoformans* determines the progression of the disease.

## 2. Invasion of *C. neoformans* into the Central Nervous System

As a neurotropic pathogen, *C. neoformans* invades the brain and causes meningoencephalitis, which represents the main clinical manifestation of cryptococcosis. Two relevant entry sites have been described for neurotropic pathogens including viruses and bacteria: the BBB and the blood–cerebrospinal fluid barrier (BCSFB) [19]. *C. neoformans* has demonstrated limited ability to migrate through the choroid plexus to invade the CNS, based on evidence that the choroid plexus remains normal and free of fungal cells in infected mice [20,21]. Furthermore, there have been limited clinical cases of choroid plexitis reported in human patients [22,23,24,25], which supports the idea that crossing through the BCSFB plays a minor role during cryptococcal dissemination into the CNS. Instead, cryptococcal clusters have been often observed next to the cortical microvasculature of the brain and cerebellum [20,21], demonstrating that *C. neoformans* crosses the BBB for brain invasion.

**Figure 1:**
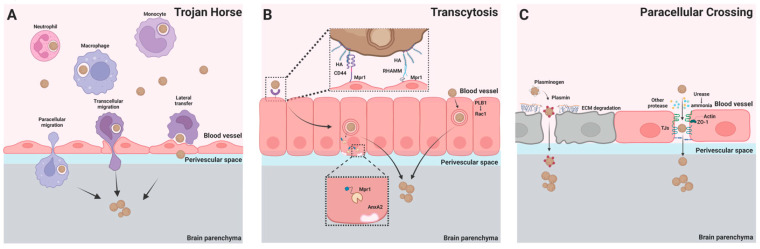
*C. neoformans’* transmigration to the brain across the BBB. Three pathways have been proposed for the BBB crossing of *C. neoformans*. (**A**) Trojan horse. Phagocytes can carry ingested *C. neoformans* across the BBB through paracellular and transcellular migration. Phagocytes containing *C. neoformans* can also directly transfer the fungal cell to brain endothelial cells through lateral transfer, leading to BBB crossing of the organism. (**B**) Transcytosis. Interactions of cryptococcal hyaluronic acid with endothelial CD44 or receptor of hyaluronan-mediated motility (RHAMM) lead to endocytosis of the yeast cell. Following endocytosis, cryptococcal Mpr1 interacts with Annexin A2 (AnxA2) to facilitate exocytosis of the organism from the endothelial cells. In addition, cryptococcal phospholipase B1 (PLB1) promotes transcytosis through activation of host cell Rac1. (**C**) Paracellular crossing. Host plasminogen (white) binds to *C. neoformans* and is converted to the serine protease plasmin (red). Plasmin degrades the extracellular matrix (ECM), facilitating fungal crossing of the BBB. The tight junction of the BBB can be damaged by cryptococcal urease (due to the toxic effects of urease-induced ammonia) and other secreted cryptococcal proteases. *C. neoformans* can invade the brain across the damaged BBB.

The BBB is a selectively permeable barrier that separates the brain parenchyma from the vascular compartment [26]. The major components of the BBB are endothelial cells, pericytes, and astrocytes [27]. Endothelial cells line the lumen side of the brain vasculature and are connected by tight junctions. During inflammation, circulating leukocytes can transmigrate into the brain parenchyma paracellularly through the tight junction or transcellularly through endocytosis mediated by the endothelial cells [28]. To enter the brain, *C. neoformans* must cross the BBB, and three major pathways have been proposed for crossing the BBB including Trojan horse, transcytosis, and paracellular crossing [29,30,31] (Figure 1).

### 2.1. Trojan Horse

*C. neoformans* is a facultative intracellular pathogen that can survive and grow inside phagocytes [32]. The expulsion of live *C. neoformans* (known as vomocytosis) by macrophages has been observed in vitro and in vivo following phagocytosis of the fungus [33,34,35]. In addition, *C. neoformans* can move directly from infected to uninfected macrophages [36,37]. These unique properties of *C. neoformans* support the hypothesis that phagocytes contribute to the spread and dissemination of *C. neoformans* from the infected lung to the CNS. Indeed, mononuclear phagocytes harboring *C. neoformans* have been detected in the perivascular space of the brain [38,39]. The deletion of alveolar macrophages or CD11c^+^ cells (alveolar macrophage and dendritic cells (DCs)) in the lungs significantly reduced the dissemination of *C. neoformans* to the lung lymph nodes and the brain [40,41], suggesting a role for these cells in extrapulmonary dissemination. In addition, the intravenous transfer of *C. neoformans*-infected macrophages to recipient mice enhanced brain colony-forming unit (CFU) levels [42,43], while the depletion of monocytes decreased brain CFU levels [39,42], providing further evidence that phagocytes are capable of transporting fungal cells to the CNS.

Using a monolayer of human cerebral microvascular endothelial cells (hCMEC/D3) cultured in vitro, monocytes infected with *C. neoformans* were directly seen to cross brain endothelial cells [44,45], demonstrating that these cells can facilitate neuroinvasion. Supporting this, intravital microscopy revealed that a substantial number of CX3CR1^+^Ly6C^low^ monocytes were recruited to the brain microvasculature 12 h after *C. neoformans* infection and were seen to engulf *C. neoformans*, adhere to the luminal wall of the brain microvasculature, and transmigrate to the parenchyma [46]. In contrast, CCR2^+^Ly6C^hi^ monocytes were observed to accumulate in the brain starting 14 days post intravenous *C. neoformans* infection, and instead drive brain inflammation in mice as well as humans [47,48].

Further support of the Trojan horse mechanism of brain invasion by *C. neoformans* comes from clinical studies demonstrating that the efficient internalization of *C. neoformans* by phagocytes positively correlated with CSF fungal burdens and the risk of death in HIV-associated cryptococcosis patients [49]. Moreover, monocyte-derived macrophages from HIV/AIDS patients displayed a higher phagocytosis efficiency of *C. neoformans* along with more intracellular fungal growth compared to those of health individuals [50]. With the use of miRNA transcriptomics, it has been recently shown that the cytoskeleton and myocilin, encoded by *MYOC*, were involved in cryptococcal brain dissemination by modulating the Trojan horse pathway in mice and macaques, and provide a possible explanation for higher incidences of cryptococcal meningoencephalitis in HIV/AIDS patients, who often have dysfunctional immune cell cytoskeletons [50].

### 2.2. Transcytosis

In a murine model of *C. neoformans* infection, fungal cells were observed inside endothelial cells of the brain, suggesting that *C. neoformans* invades the brain through transcytosis [38]. In vitro studies using a monolayer of human brain microvascular endothelial cells (HBMECs) have shown that *C. neoformans* can adhere to endothelial cells and cross HBMEC monolayers via a transcellular pathway [21]. Studies have shown that the cryptococcal *CPS1* gene encodes a hyaluronic acid synthase and that its product, hyaluronic acid, is critically involved in the adhesion of *C. neoformans* to brain endothelial cells [51]. It was further demonstrated that the adhesion of *C. neoformans* to HBMEC monolayers and the subsequent transcytosis was mediated by interactions between cryptococcal hyaluronic acids and CD44 expressed on HBMECs [52] and involved protein kinase C-alpha [53]. Moreover, *C. neoformans* enhanced the activity of EphA2, a tyrosine kinase receptor, through CD44, facilitating fungal migration into the brain [54]. In support of these in vitro observations, mice deficient in CD44 displayed lower brain fungal burdens following *C. neoformans* infection [55]. Despite this, CD44-deficient mice still had *C. neoformans* present in their brains, and in fact another hyaluronic acid receptor, RHAMM (receptor of hyaluronan-mediated motility), which is present in CD44-deficient mice, was also found to mediate the association of *C. neoformans* cells with mouse brain microvascular endothelial cells (BMECs) [55]. Interestingly, the brain has high levels of the sugar inositol, and inositol acquisition by *C. neoformans* leads to the upregulation of the cryptococcal *CPS1* gene and the higher production of hyaluronic acids, thus promoting the adherence and transcytosis of *C. neoformans,* which may explain *C. neoformans’* predilection for the brain [56].

Apart from hyaluronic acids, cryptococcal phospholipase B1 (PLB1) has been shown to promote the transcytosis of *C. neoformans* in cultured HBMECs through the activation of host cell Rac1 and its association with STAT3 [57]. In addition, a secreted metalloprotease (Mpr1) of *C. neoformans* was reported to be required for crossing hCMEC/D3 monolayers by interacting with cytoskeleton-endocytosis-associated protein Annexin A2; consequently, mice infected with a strain of the fungus lacking the gene encoding Mpr1 survived longer due to reduced brain fungal burdens [58,59]. Recently, an approach based on flow cytometry to quantitatively analyze fungal migration into the brain has been established, and using this approach, it has been confirmed that the internalization of *C. neoformans* by brain endothelial cells occurs in vivo [60]. Interestingly, quantitative analysis revealed that the brain endothelial cells were invaded by *C. neoformans* at a higher rate compared to *C. deneoformans*, which may reflect the higher virulence of *C. neoformans* during brain infection in mice [60].

### 2.3. Paracellular Crossing

In addition to the Trojan horse and transcytosis pathways, it has been proposed that *C. neoformans* can migrate into the brain through paracellular crossing between brain endothelial cells and across damaged endothelial barriers [30,31]. Early studies have shown that there was vessel damage and leakage at fungal arrest sites [20]. In addition, there is evidence that *C. neoformans* induces alterations of the tight junction and the cytoskeleton of endothelial cells and ultimately induces endothelial cell necrosis [61,62,63]. In this context, the cryptococcal capsule and secreted enzymes may contribute to the damage of tight junction and endothelial cells [31]. For example, cryptococcal urease is a major virulence factor and promotes fungal invasion into the CNS [29,64,65,66], likely by damaging tight junctions due to the toxic effects of urease-induced ammonia [66]. *C. neoformans* has also been found to secrete a number of proteases, which have been shown to disrupt the BBB during brain infection [67,68]. In addition, *C. neoformans* is able to bind and activate host plasminogen, leading to the conversion of plasminogen to the serine protease plasmin, which is also capable of degrading the BBB [69,70].

## 3. Host Immune Responses to *C. neoformans* in the Brain

**Figure 2:**
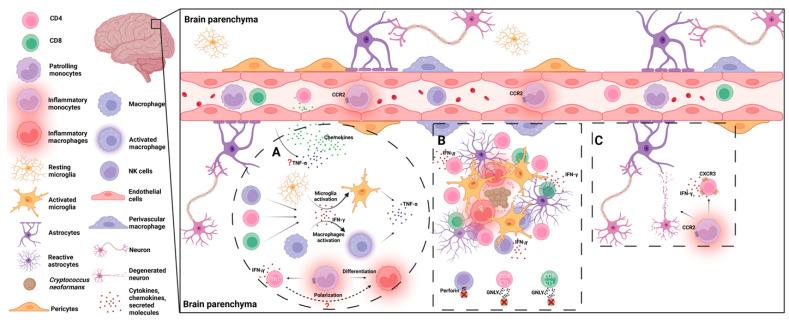
Host immune responses to *C. neoformans* in the brain. (**A**) Following brain infection of *C. neoformans*, TNF-α and chemokines are produced, facilitating leukocytes’ migration to the brain parenchyma. IFN-γ produced by CD4^+^/CD8^+^ T cells and NK/NKT cells promotes the activation of microglia and macrophages. Activated microglia and macrophages secrete TNF-α, further driving brain inflammation. Recruited inflammatory monocytes differentiate into inflammatory macrophages and help shape Th1 immune responses, which are required for fungal clearance. (**B**) As the resident glial cells, microglia and astrocytes are the first cells to respond to invading yeast cells. Later, recruited leukocytes including CD4^+^/CD8^+^ T cells, NK/NKT cells, and inflammatory monocyte/macrophages accumulate in and around the fungal clusters. As effector cells, microglia and macrophages internalize the fungal cells. T cells and NK cells secrete IFN-γ to promote fungicidal activity of these phagocytes. NK cells and T cells are also involved in direct killing of the fungal cells through release of perforin or granulysin (GNLY). (**C**) Inflammatory responses are required for fungal clearance in the brain but must be tightly controlled. Inflammatory monocytes and IFN-γ-secreting CXCR3^+^ CD4^+^ T cells facilitate neuronal damage and cause immunopathology during cryptococcal meningoencephalitis.

Following its invasion into the brain, *C. neoformans* cells begin to grow. *C. neoformans* will first encounter microglia, the macrophages residing in the brain. Interactions between microglia and invading *C. neoformans* lead to neuroinflammation including the production of proinflammatory cytokines and chemokines, which promotes the recruitment and accumulation of innate immune cells as well as adaptive immune cells. These immune cells, along with the cytokines secreted by them, are critically involved in fighting the fungal cells [9,13] (Figure 2).

### 3.1. Microglia

Tissue-resident macrophages are a diverse population of leukocytes that reside in mammalian tissues and play a prominent role in tissue homeostasis and host defense [71]. Tissue-resident macrophages are usually embryonic in origin and self-renewing under steady-state conditions [72,73]. As brain-resident macrophages, microglia are found throughout the brain parenchyma and play a central role in immune responses in the brain. During their resting state, microglia extend their dendrites to survey the brain [74]. Upon activation, microglia enlarge their cell bodies and adopt an “amoeboid” morphology with thicker ramifications and can engulf and clear dead neurons, pathogens, and pathogen-infected cells [75,76,77,78,79,80].

During a brain infection with the fungal pathogen *Candida albicans*, CARD9^+^ microglia mediate protection by promoting neutrophil recruitment in a IL-1β- and CXCL1-dependent manner [81]. Studies on the interactions between microglia and *C. neoformans* revealed that microglia can internalize *C. neoformans* in vitro [82,83,84,85,86] as well as in the human brain [87]. The internalization of nonopsonized *C. neoformans* by swine microglia was reported to involve the CD14 receptor [84]. The presence of capsule-binding antibodies was shown to enhance the phagocytosis of *C. neoformans* by human microglial cells [85], and in the presence of anti-cryptococcal antibodies, *C. neoformans*-stimulated microglia secreted proinflammatory chemokines MIP-1α and MIP-1β [88]. However, the cryptococcal capsule has been found to inhibit phagocytosis by murine microglial cells [86], as well as down-regulate the production of MIP-1α and MIP-1β [88].

The stimulation of microglia by TLR agonists also promotes phagocytosis and the killing of *C. neoformans* and is associated with enhanced secretions of proinflammatory cytokines including TNF-α [83]. In addition, the killing of *C. neoformans* by microglia was positively correlated with nitric oxide secretion [89,90]. Interestingly, the intracellular iron load of microglia, rather than the stimulation by IFN-γ, enhanced the anticryptococcal activity of microglia in vitro [91,92]. As a facultative intracellular pathogen, *C. neoformans* can also survive and proliferate within microglial cells depending on the activation status of the microglia [87,93].

Murine studies showed that MHC class II-positive perivascular microglial cells were involved in resistance to *C. neoformans* brain infection [94]. In line with this report, immunotherapy with a combined administration of anti-CD40 and IL-2 during a *C. neoformans* infection reduced fungal burdens in the brain, which correlated with an increase in MHC class II expression in microglia in an IFN-γ-dependent manner [95,96]. More recently, it has been shown that the number of microglial cells is significantly enhanced following the upregulation of MHC class II and CD11c in the cells during a brain infection with *C. neoformans* [97].

### 3.2. Inflammatory Monocytes/Macrophages

Monocytes consist of two major populations. These include CD14^hi^CD16^−^ and CD14^low^CD16^hi^ monocytes in humans, and the corresponding CCR2^hi^CX3CR1^low^Ly6C^hi^ inflammatory monocytes and CCR2^low^CX3CR1^hi^Ly6C^low^ monocytes in mice [98,99,100]. During infection and inflammation, Ly6C^hi^ inflammatory monocytes migrate from the bone marrow to the periphery blood through CCR2 signaling and are recruited to the infected or inflamed tissues [98,101]. The recruited Ly6C^hi^ inflammatory monocytes can differentiate to monocyte-derived Ly6C^hi^CD11b^+^ inflammatory macrophages and DCs [98]. These recruited Ly6C^hi^ mononuclear phagocytes (i.e., Ly6C^hi^ monocytes and their derivatives) secrete high levels of proinflammatory cytokines, promote inflammation, and act as effector cells and play an essential role in the clearance of pathogens [98].

As the signaling of CCR2 with its ligands, including CCL2 and CCL7, is required for the emigration of Ly6C^hi^ monocytes from the bone marrow, CCR2^−/−^ mice have been widely used to study the functions of Ly6C^hi^ inflammatory monocytes and macrophages [98,101]. Previous studies have shown that Ly6C^hi^ inflammatory monocytes and macrophages display a prominent role in the clearance of various fungal pathogens in the lungs, including *Aspergillus fumigatus* [102,103], *Candida albicans* [104], *Histoplasma capsulatum* [105,106], and *Blastomyces dermatitidis* [105].

During a pulmonary infection with *C. neoformans*, the disruption of CCR2 signaling led to higher fungal burdens and coincided with the development of detrimental Th2 responses in the infected lung [107,108]. CCR2 signaling promoted Ly6C^hi^ inflammatory monocyte migration to the infected lung, leading to the differentiation and accumulation of Ly6C^hi^ inflammatory macrophages and DCs [109,110,111]. These effector cells secrete nitric oxide and TNF-α and have been shown to kill *C. neoformans* in the lung [109]. Moreover, recent studies showed that Ly6C^hi^ inflammatory monocytes and macrophages are critically involved in the protection generated by candidate vaccine strains of *C. neoformans* [112,113,114,115]. However, the fungicidal activity of Ly6C^hi^ inflammatory monocytes and macrophages must be tightly controlled to avoid tissue destruction [98]. In this regard, more recent studies have shown that an enhanced accumulation of Ly6C^hi^ inflammatory monocytes and macrophages mediated detrimental immune responses in the lung in a murine model of an acute infection with *C. neoformans* [116,117].

CCL2 is expressed by microglia, astrocytes, and endothelial cells in the brain under physiological conditions [118]. An internal crosstalk between inflammatory monocytes with microglia was observed during West Nile Virus-induced encephalitis [119], although whether it occurs during cryptococcal meningoencephalitis remains unknown. Clinical studies have shown that inflammatory monocytes are recruited to the CNS of human patients during cryptococcal meningitis [47,48]. The impaired recruitment of leukocytes to the brain in MIP-1α knockout mice correlated with an impaired clearance of *C. neoformans* in the brain [120], suggesting that the accumulation of leukocytes including inflammatory monocytes in the brain is required for fungal clearance. However, the brain’s inflammatory responses mediated by inflammatory monocytes/macrophages and other leukocytes must be tightly controlled to avoid neuropathology during brain infection with *C. neoformans* [97,121].

### 3.3. NK/NKT Cells

Natural killer cells (NK cells) are innate immune cells and are well known to have the capability of killing virus-infected cells and tumor cells. There are limited NK cells in the brain parenchyma under steady-state conditions, but NK cells have been shown to be recruited in a CX3CR1-dependent manner to glioblastomas, including both common and aggressive brain tumors. Both resident and recruited NK cells are activated by the cytokines IL-2, IL-15, and IL-19 to produce TNF-α and IFN-γ, as well as perforin and granzymes, which are able to lyse target cells [122]. Early studies have shown that NK cells can bind to *C. neoformans* and directly kill the fungus [123,124,125,126,127]. It was later found that NK cells use perforin but not granulysin to kill *C. neoformans* [128] and that this requires PI3K-dependent ERK1/2 signaling [129]. It was further demonstrated that β-1,3-glucan, a component of the fungal cell wall, binds to NKp30 expressed on NK cells, thereby mediating the recognition and killing of *C. neoformans* [130,131]. In addition to the direct killing of *C. neoformans*, NK cells can secrete IFN-γ upon stimulation with IL-12 and IL-18 to activate phagocytes such as macrophages, leading to the indirect killing of *C. neoformans* [132,133,134].

NKT cells are a unique subset of T lymphocytes and can be identified by their expression of both T cell receptors (TCR) along with NK cell lineage receptors. Invariant NKT (iNKT) cells reside in the brain parenchyma during homeostasis [135]. While iNKT cells are CD1d-restricted T cells, both the recruitment and activation of peripheral NKT cells to the CNS are CD1d-independent [136]. iNKT cells accumulate in the lung in an MCP-1-dependent manner during a pulmonary cryptococcal infection and contribute to the development of protective Th1 immune responses [137]. It was reported that α-Galactosylceramide-activated NKT cells enhanced their secretions of IFN-γ in the absence of IL-18 signaling during systematic cryptococcal infection [138]. Aged C57BL/6 mice with enhanced mature NKT cells displayed stronger fungal resistance in the lungs, indicating that NKT cells play a role in mediating protection [139]. However, their role during cryptococcal CNS infection has not yet been elucidated.

### 3.4. CD4/8^+^ T Cells

Following antigen presentation, naïve T cells proliferate and differentiate to antigen-specific T cells, which migrate to infected tissues. Cryptococcal meningoencephalitis primarily affects immunocompromised hosts such as HIV/AIDS patients whose CD4^+^ T cell responses are impaired [1,140], demonstrating the essential role of T cells in host defense against *C. neoformans* infection.

During a pulmonary infection with *C. neoformans*, both CD4^+^ and CD8^+^ T cells were substantially recruited to the lung, and the depletion of either CD4^+^ or CD8^+^ T cells prevented pulmonary clearance and resulted in a significant colonization of the brain in mice, demonstrating that both CD4^+^ and CD8^+^ T cells are required to clear *C. neoformans* infection [141,142,143,144]. The depletion of either CD4^+^ or CD8^+^ T cells markedly reduced the influx of myeloid cells, including monocytes and neutrophils, to the infected lung [142]. Interestingly, the depletion of CD4^+^ T cells did not affect the influx of CD8^+^ T cells to the infected lung [141,142,143] and CD8^+^ T cells functioned independently of CD4^+^ T cells to control fungal growth by limiting the survival of *C. neoformans* within macrophages through IFN-γ production [142,145].

During a brain infection with *C. neoformans*, a substantial accumulation of CD4^+^ T cells, and to a lesser extent CD8^+^ T cells, was observed in the brain at weeks 3 and 4 post-infection [97]. Following a cryptococcal infection, chemokines such as CCL2, CXCL1, CCL5, and CXCR3 ligands (CXCL9, 10, and 11) were secreted by astrocytes and T cells were recruited into the brain in a CXCR3-dependent manner [146]. The in vivo depletion of CD4^+^ T cells markedly reduced leukocyte accumulation in the brains of immunized mice and was associated with an exacerbated CNS infection [147]. Another study showed that CD4^+^ T cells were important for the optimal infiltration of inflammatory cells into the brain and were required for optimal regional IFN-γ secretion and iNOS expression in the *C. neoformans*-infected brains of immunized mice [148]. However, recent studies have shown that the depletion of CD4^+^ T cells prevented brain immunopathology and significantly enhanced the survival of mice despite enhanced fungal growth during a brain infection with *C. neoformans* [97]. It was further demonstrated that CXCR3^+^ CD4^+^ T cells mediated lethal brain pathology without contributing to fungal clearance in the brain during cryptococcal meningoencephalitis [146], emphasizing the importance of immune balance in the CNS.

In addition to regulating immune responses, T cells have been shown to bind to *C. neoformans* and directly kill the fungus. Human peripheral CD8^+^ T cells were found to use granulysin to kill *C. neoformans* in vitro, and this fungicidal activity was dependent on CD4^+^ T cells and IL-15 [149]. Likewise, cytotoxic CD4^+^ T cells mediated the killing of *C. neoformans* using granulysin and the activation of this pathway was defective in HIV^+^ patients [150]. Moreover, the expression of granulysin by cytotoxic CD4^+^ T cells required the PI3K- and STAT5-dependent expression of IL-2Rβ, which is also defective in HIV-infected patients [151].

### 3.5. Proinflammatory Cytokines

Cytokines play critical roles in regulating immune responses to *C. neoformans* [9]. Th1 immune responses, characterized by IFN-γ secretion and the classical activation of macrophages, are required to control *C. neoformans* infection [112,152,153,154]. In contrast, Th2 responses, characterized by IL-4, IL-5, and IL-13 production, with alternative macrophage activation worsen the disease [155,156,157,158]. As a signature cytokine of Th1 responses, IFN-γ plays a central role in combating *C. neoformans* infection. Early studies showed that IFN-γ activation of macrophages was critical for anticryptococcal activity [159,160,161]. During a *C. neoformans* pulmonary infection, IFN-γR^−/−^ mice displayed significantly higher fungal burdens in the lungs and were markedly more susceptible to *C. neoformans* infection than wild-type mice [152,162]. Interestingly, the infection of mice with a murine gamma interferon-producing *C. neoformans* strain completely protected the mice from infection with wild-type *C. neoformans* [163].

During a brain infection with *C. neoformans*, a substantial amount of IFN-γ was detected in the brains of mice [97,164]. The in vivo neutralization of IFN-γ exacerbated a cryptococcal CNS infection [147,165]. During an intracranial infection of *C. neoformans*, IFN-γ was shown to mediate protection by activating microglial cells in the brains of mice [96]. Human astrocytes activated by IFN-γ and IL-1β in vitro inhibited cryptococcal growth by a nitric oxide-mediated mechanism [166]. In clinical settings, higher secretions of IFN-γ by CD4^+^ T cells were associated with an improved survival of patients [167] and the addition of IFN-γ to standard treatments significantly enhanced the rate of clearance of *C. neoformans* infection from the CSF during cryptococcal meningoencephalitis [168,169].

Th17 responses, characterized by IL-17A, also contribute to anti-cryptococcal immunity [9]. The development of Th1/Th17 responses and the classical activation of macrophages resulted in a significant containment of *C. neoformans* cells in the lungs of mice [170]. Using IL-17A^−/−^ mice, it was demonstrated that IL-17A mediated protection through the promotion of leukocyte recruitment, activation, and IFN-γ secretions [171]. It was recently reported that a type I IFN induction via poly-ICLC protected mice against cryptococcosis and that the protective effect was diminished by the neutralization of IL-17A, demonstrating the protective function of IL-17A [172].

TNF-α is another cytokine that mediates protection during cryptococcosis [9]. Neutralization of TNF-α reduced leukocyte influx to the lung and resulted in higher fungal burdens during a pulmonary infection of *C. neoformans* [173]; TNF-α was required for the induction of IL-12 and IFN-γ, which mediated protection during a pulmonary cryptococcal infection [174]. Moreover, TNF-α mediated protection in a lung infected with *C. neoformans* by inducing DC1 polarization and the initial Th1/Th17 responses at the early stages of infection [175]. TNF-α expression by an engineered *C. neoformans* strain led to protective immune responses during pulmonary cryptococcosis [176]. The enhanced secretion of TNF-α was detected in the brain during a *C. neoformans* infection [164]. During a brain infection with *C. neoformans*, the neutralization of TNF-α led to a marked increase of brain fungal burdens, indicative of the anti-fungal activity of TNF-α in cryptococcal meningoencephalitis [165]. However, the mechanism of the protective role of TNF-α during a cryptococcal CNS infection has not been elucidated.

## 4. Fungal Virulence Factors as Immune Modulators

**Figure 3:**
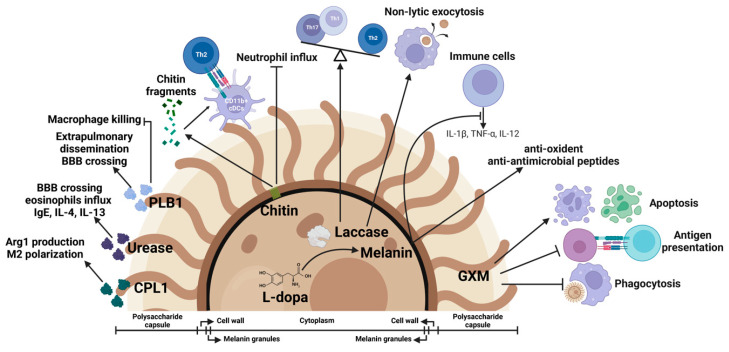
Cryptococcal virulence factors as immune modulators. Fungal virulence factors mediate cryptococcal pathogenesis and function as host immune modulators. Glucuronoxylomannan (GXM) from the polysaccharide capsule inhibits phagocytosis of *C. neoformans* by macrophages, suppresses antigen presentation, and induces apoptosis of macrophages and T cells. Melanin protects the yeast cells from oxidants and antimicrobial peptides. Melanization suppresses the production of cytokines such as IL-1β, TNF-α, and IL-12. Laccase, an enzyme required for biosynthesis of melanin, is involved in regulation of nonlytic exocytosis of *C. neoformans* from macrophages and contributes to the skewing of Th1/Th17 to Th2 immune responses. Chitin fragments promote Th2 responses and inhibit neutrophil influx. PLB1 promotes fungal dissemination and inhibits macrophage killing. Urease facilitates fungal brain invasion and induces Th2 responses. Secreted protein CPL1 promotes arginase-1 production and shapes the M2 polarization.

To fight against host immune defense, *C. neoformans* develops immune evasion strategies. The major virulence factors of *C. neoformans* include its polysaccharide capsule, melanin production, and the secretion of extracellular enzymes [18]. These virulence factors not only contribute to cryptococcal pathogenesis but are also involved in the modulation of host immune responses [177] (Figure 3).

### 4.1. Polysaccharide Capsule

One of the major virulence factors for *C. neoformans* is the polysaccharide capsule [18]. The capsule is located outside of the fungal cell wall and protects the fungus from immune recognition and destruction. Studies have shown that the capsule is mainly composed of two types of polysaccharides and that the dominant polysaccharide (>90% of the total capsule polysaccharides) is glucuronoxylomannan (GXM); another polysaccharide, known as galactoxylomannan (GalXM), represents the other 5-8% of the capsule along with nonpolysaccharide components such as mannoprotein [15,177,178]. The capsular polysaccharide is synthesized intracellularly and is believed to be translocated to the extracellular space within vesicles [178].

The cryptococcal capsule modulates host immune responses in multiple ways [15,177]. The capsule prevents the phagocytosis of the fungus by macrophages [32,179]. GXM is known to inhibit the antigen-presenting capacity of human monocytes and monocyte-derived macrophages through the induction of IL-10 secretion and the suppression of MHC-II expression, resulting in impaired T cell proliferation [180,181,182]. GXM also induces the apoptosis of macrophages and T cells through the induction of Fas ligand expression [183,184] or the induction of iNOS expression and NO production in a caspase-independent pathway [185]. GXM also inhibits DC activation and maturation [186,187]. Of note, the regulated release of capsular GXM of *C. neoformans* suppresses leukocyte migration to the brain, promoting fungal growth in the brain [188]. In clinical settings, capsule shedding (exocellular GXM) can be detected in the serum and CSF of patients during cryptococcal meningitis [189,190]. There is a positive correlation between serum GXM titers and mortality during HIV-associated cryptococcal meningitis [191]. Moreover, an increased size of the capsule correlated with impaired fungal clearance and reduced CSF leukocytes and proinflammatory cytokines including IL-6 and IFN-γ, confirming the immunosuppressive properties of the capsule in HIV-associated cryptococcal meningitis [189].

### 4.2. Laccase Activity and Melanin Formation

In addition to the capsule, laccase and melanin are known major virulence factors of *C. neoformans* [18,192,193,194,195,196]. Laccase is required for the biosynthesis of melanin [197], which accumulates in the *C. neoformans* cell wall [18]. The published work suggested that laccase facilitated the extrapulmonary dissemination of *C. neoformans* [195]. This is likely, at least in part, because laccase activity protected *C. neoformans* from being killed by alveolar macrophages [193]. Moreover, laccase activity enhanced pulmonary eosinophilia and shifted immune polarization from protective Th1/Th17 and M1 responses to deleterious Th2 and M2 responses, promoting fungal growth during a pulmonary infection with *C. neoformans* [196]. Recently, it has been shown that laccase activity dampened Th17 responses and neutrophil accumulation and function during the early stages of an infection with *Cryptococcus gattii* (*C. gattii*) [198]. Interestingly, laccase is also critically involved in the regulation of the nonlytic exocytosis of *C. neoformans* from macrophages [199]. In HIV-associated cryptococcosis patients, cryptococcal laccase activity correlated with fungal survival and poor fungal clearance in CSFs [49], arguably through the regulation of the interaction between *C. neoformans* and phagocytes. Thus, cryptococcal laccase modulates host defenses through multiple mechanisms involving innate and adaptive immunity.

Located in the cell wall, cryptococcal melanin is a powerful antioxidant and helps protect fungal cells against oxygen- and nitrogen-derived oxidants generated by host effector cells [17,200,201,202]. Early studies have shown that the melanization of *C. neoformans* correlated with higher levels of IL-4 and MCP-1 and enhanced leukocyte recruitment and interfered with phagocytosis, which is indicative of immunomodulation [203]. Due to the presence of melanin, *C. neoformans* is less susceptible to the cationic antimicrobial peptides released by phagocytes [204]. Solubilized melanin has been recently shown to inhibit macrophage functions [205]. In addition to modulating innate immunity, melanin inhibited antigen recognition and down-regulated T cell immunity and inflammation during a *C. neoformans* pulmonary infection [206]. During a brain infection with *C. neoformans*, melanization suppressed the production of IL-12, IL-1β TNF-α, IFN-γ, and iNOS, promoting mortality [207].

### 4.3. Phospholipase B1 Activity

Phospholipases are extracellular enzymes that degrade cell membrane phospholipids [18]. Amongst them, phospholipase B1 (PLB1) has been well-characterized and is known as one of the major virulence factors of *C. neoformans* [18,43,208,209,210,211,212,213]. The depletion of PLB1 significantly reduced fungal burdens during a pulmonary infection with *C. neoformans* [211]. In addition, PLB1 is essential for the extrapulmonary dissemination of *C. neoformans* [212,213] and the transmigration of the fungus across the BBB [43,57]. Importantly, PLB1 is required for the release of arachidonic acid from phospholipids and the production of cryptococcal eicosanoids, which down-regulates macrophages’ functions in vitro and during a pulmonary infection [213]. In line with this finding, recent work suggested that PLB1 promoted the proliferation of *C. neoformans* within macrophages and inhibited the killing of the fungus within the phagosome [214].

### 4.4. Urease Activity

Urease is an extracellular enzyme and is considered a major virulence factor of *C. neoformans* [64]. *C. neoformans* secretes urease, which catalyzes the hydrolysis of urea to ammonia and carbamate [18]. The published work suggests that urease activity promotes cryptococcal brain invasion [29,65,66]. In addition, an infection with urease-producing *C. neoformans* correlated with enhanced eosinophil influx; higher levels of IgE, IL-4, and IL-13; and, alternatively, the activation of macrophages, suggesting that urease activity promotes Th2 immune responses [215].

### 4.5. Chitin

Chitin is an essential component of the cell wall of *C. neoformans,* and the fungus has eight putative chitin synthases [216]. Mammalian hosts possess chitotriosidase, an enzyme that can degrade chitin into chitin fragments [217,218]. Early studies showed that degraded chitin fragments could trigger human macrophage activation [219]. In a murine model of an infection with *C. neoformans*, the host’s chitotriosidase degraded fungal chitin into small fragments that led to Th2 differentiation by conventional CD11b^+^ DCs, demonstrating that chitin recognition via chitotriosidase promoted detrimental Th2 immune responses [116]. Of note, chitotriosidase activity correlated with cryptococcal infection in humans [116]. In contrast to the negative impact of chitin on immune responses, it has been recently reported that mice infected with *C. neoformans* depleted of chitin synthase 3 died significantly earlier due to an excessive neutrophil influx, demonstrating a beneficial role of chitin in preventing lethal immune responses to *C. neoformans* [220].

### 4.6. CPL1

CPL1 is a secreted cryptococcal protein encoded by CNAG_02797/*CPL1* [221]. A recent study showed that CPL1 induced arginase-1 production in macrophages and enhanced macrophage sensitivity to IL-4 signaling through the activation of TLR4 signaling and the promotion of the phosphorylation of STAT3. As a result, CPL1 drove the alternative activation of macrophages and promoted harmful type 2 immunity during cryptococcosis [222].

## 5. Concluding Remarks

Although a cryptococcal infection starts in the lungs, cryptococcosis commonly presents as meningoencephalitis [3]. Cryptococcal meningoencephalitis is among the most devastating complications of HIV/AIDS patients and is a leading cause of mortality among HIV/AIDS patients [223,224]. One of the critical steps to causing the illness is fungal dissemination and invasion into the brain by crossing the BBB [31]. A fungal invasion leads to host defenses including innate and adaptive immunity involving resident microglial cells and recruited myeloid cells and T cells [13]. In response to host defense, *C. neoformans* uses multiple virulence factors to suppress host immune responses [177]. In the past decades, much progress has been achieved with respect to understanding the interaction between hosts and *C. neoformans*. However, many questions remain unanswered. For example, which pathway is dominant during a brain invasion by *C. neoformans*? How do microglial cells interact with recruited leukocytes in the brain during a *C. neoformans* infection? What is the strategy that the fungus uses to survive and replicate in the brain? By addressing these questions, we will have a better understanding of the complex interaction between the host and *C. neoformans*.
